# Influence of parallel pins on the angle deviation for placement of dental implants: an in vitro study

**DOI:** 10.1186/s12903-024-03883-w

**Published:** 2024-01-26

**Authors:** Héctor González Menéndez, Juan Lorrio Castro, Paulina Rodríguez Torres, Susana de la Vega Buró, Álvaro Zubizarreta-Macho, Elena Riad Deglow, Ana Belén Lobo Galindo, Sofía Hernández Montero

**Affiliations:** 1 Department of Implant Surgery, Faculty of Health Sciences, Alfonso X El Sabio University, 28691 Madrid, Spain; 2 Department of Surgery, Faculty of Medicine and Dentistry, University of Salamanca, 37008 Salamanca, Spain

**Keywords:** Dental implants, Implantology, CBCT, Angle deviation, Parallel pin

## Abstract

The aim of the present study was to analyze and compare the angle deviation of two, four and six adjacent dental implants placed with and without straight parallel pins. Materials and Methods: Two hundred and forty (240) dental implants were selected and randomly allocated into the following study groups: Two dental implants placed with straight parallel pins (Ref.: 144-100, BioHorizons, Birmingham, AL, USA) (*n* = 10) (2PP); Two dental implants placed without parallel pins (*n* = 10) (2withoutPP); Four dental implants placed with straight parallel pins hT(*n* = 10) (4PP); Four dental implants placed without parallel pins (*n* = 10) (4withoutPP); Six dental implants placed with straight parallel pins (*n* = 10) (6PP) and Six dental implants placed without parallel pins (*n* = 10) (6withoutPP). The dental implants randomly assigned to groups 2PP and 2withoutPP were placed into standardized polyurethane models of partially edentulous upper jaws in tooth positions 2.4 and 2.6, the dental implants randomly assigned to groups 4PP and 4withoutPP were placed into standardized polyurethane models of fully edentulous upper jaws in tooth positions 1.6, 1.4, 2.4 and 2.6, and the dental implants randomly assigned to groups 6PP and 6withoutPP were placed into standardized polyurethane models of fully edentulous upper jaws in tooth positions 1.6, 1.4, 1.2, 2.2, 2.4 and 2.6. Afterwards, postoperative CBCT scans and digital impressions were aligned in a 3D implant-planning software to compare the angle deviation (°) of two, four and six adjacent dental implants placed with and without straight parallel pins using the General Linear Model statistical analysis. Results: Statistically significant differences were found between the angle deviation of 2 dental implants placed with straight parallel pins (*p <* 0.0001) and between the angle deviation of 4 dental implants placed with straight parallel pins (*p =* 0.0024); however, no statistically significant differences were found in the angle deviation of 6 dental implants placed with straight parallel pins (*p =* 0.9967). Conclusion: The use of a straight parallelization pin results in lower angle deviation between two and four adjacent dental implants; however, it is not effective for a larger number of dental implants.

## Introduction

A favorable dental implant position is always desirable, since it allows a homogeneous occlusal load distribution, favorable aesthetics, improves the accuracy of both conventional and digital impressions, the prosthetic fabrication procedures, the passive adjustment of implant-supported restorations and their subsequently biological complications such as bone loss [1]. In addition, Dario LJ suggested that parallel dental implant placement may be impossible in curved edentulous arch, especially with a facial concavity [2]; therefore, computer-aided implant systems have been developed in order to allow a more predictable and safer dental implant placement and prosthetic restoration, than freehand technique for the dental implant placement. Computer-aided surgery using static navigation systems has been widely used for the dental implant placement showing a mean horizontal deviation of 1.2 mm at the coronal entry point, 1.4 mm at the apical end point, and an angular deviation of 3.5° [3] and computer-aided surgery using dynamic navigation systems has shown lower mean horizontal deviations at the coronal entry point (0.71 ± 0.40 mm), apical end point (1.00 ± 0.49 mm), and angular deviation (2.26 ± 1.62°) [4]; therefore, computer-aided implant systems are not totally accurate.

Moreover, parallelism between multiple dental implants is essential on the mechanical and biological prognosis of implant-supported restorations; therefore, no-parallel dental implants can be restored through preangled or custom-angled abutments to achieve prosthetically desired parallelism between implants and to make an appropriate fabrication of implant restoration [5, 6]; however, angled abutments may result in increased stress on the dental implants and surrounding bone tissue [**Error! Bookmark not defined.**,**Error! Bookmark not defined.**]; therefore, clinicians must pay special attention during the placement of dental implants. Shepherd NJ recommend parallel pin guides to help ensure both precise location and angulation of dental implant placement, since this surgical piece can be placed into the pilot hole in the bone and check the direction of the surgical drilling respect the adjacent dental implant [7].

In addition, the dental implant angulation has demonstrated to influence the accuracy and reliability of digital impressions. Gómez-Polo et al. reported that the implant angulation significantly affects (*p* < 0.001) the outcome of complete-arch implant digital scans, specifically, the parallel implant analog position obtained better accuracy than the angulated positions [8, 9]. Moreover, Carneiro Pereira et al. reported that parallel dental implants improve prosthesis longevity and facilitate the impression making steps when compared with angled implants and concluded that digital scanning is reliable although more attention must be taken in patients where the angles between implants are greater than 15 degrees [10]. However, Alexander Hazboun et al. did not show statistically significant differences (*p* = 0.070) in the impressions made with open or closed tray technique in angulated dental implants at 0, 15, or 30 degrees [11].

Moreover, it has been also analyzed the the impact of tilted implants o the success rate and and marginal bone loss. Ata-Ali et al. reported that no evidence of differences in success rate between tilted and axial implants and a similar marginal bone loss between tilted and axial implants; therefore, it can be deduced that tilted implants exhibit the same evolutive behavior as axial implants [12].

The aim of the present study was to analyze and compare the angle deviation of two, four and six adjacent dental implants placed with and without straight parallel pins*,* with a null hypothesis (H_0_) stating that there would be no difference between the angle deviation of adjacent dental implants placed with straight parallel pins and the angle deviation of adjacent dental implants placed without straight parallel pins.

## Materials and methods

### Study design

A randomized controlled experimental trial was conducted in compliance with the principles laid out by the International Organization for Standardization (ISO 14801). The study was conducted between January and March 2022 at the Dental Centre of Innovation and Advanced Specialties at Alfonso X El Sabio University in Madrid, Spain. The Ethical Committee of the Faculty of Health Sciences at University Alfonso X El Sabio approved the study in December 2021. A power effect of 87.2 was used to determine the sample size (anything above 80 was considered acceptable). Two hundred and forty (240) conical internal hex implant-abutment connection dental implants were included in the study to ensure a power effect of 80.00% for detecting statistically significant differences. The bilateral Student’s t-test of two independent samples was used to evaluate the null hypothesis *H*_0_ : *μ*_1_ = *μ*_2_, with a significance level of 5.00%.

### Experimental procedure

Two hundred and forty (240) conical internal hex implant-abutment connection dental implants (3.8 × 10 mm, Ref.: TRX3810, BioHorizons, Birmingham, AL, USA) were placed in sixty standardized polyurethane models of partially and fully edentulous upper jaws (Sawbones Europe AB, Malmo, Sweden) modeled after on two real clinical cases. The patients gave their informed consent for their CBCT to be used in the study. These CBCT scans were used to generate the standardized polyurethane models of partially and fully edentulous upper jaws. The conical internal hex dental implants T were randomly (Epidat 4.1, Galicia, Spain) assigned to one of the following study groups: Two dental implants placed with straight parallel pins (Ref.: 144-100, BioHorizons, Birmingham, AL, USA) (*n* = 10) (2PP); Two dental implants placed without parallel pins (*n* = 10) (2withoutPP); Four dental implants placed with straight parallel pins (*n* = 10) (4PP); Four dental implants placed without parallel pins (*n* = 10) (4withoutPP); Six dental implants placed with straight parallel pins (Ref.: 144-100, BioHorizons, Birmingham, AL, USA) (*n* = 10) (6PP) and Six dental implants placed without parallel pins (*n* = 10) (6withoutPP).

**Fig. 1:**
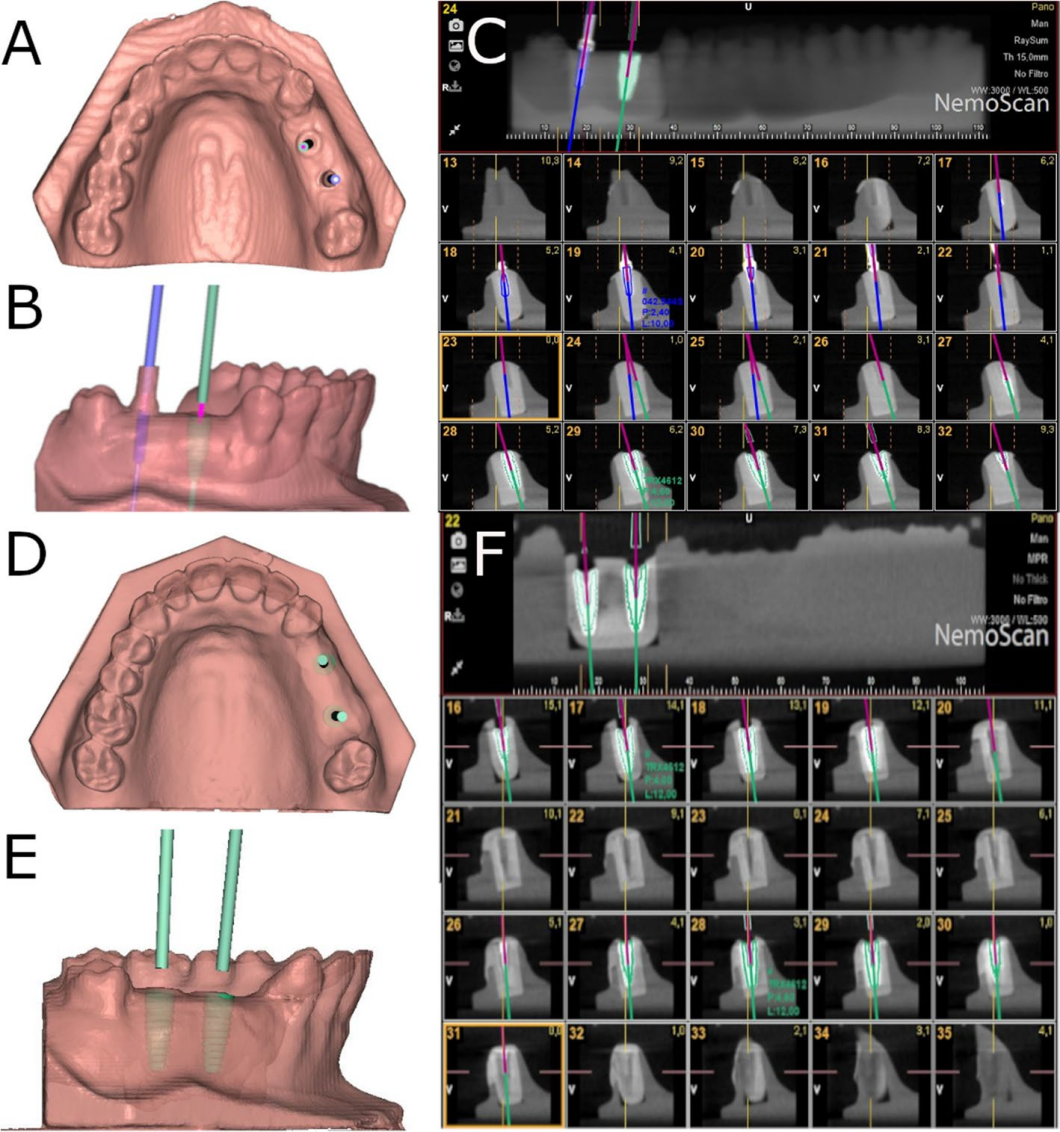
**a** Coronal, (**b**) left lateral view and (**c**) postoperative CBCT scan images of the two adjacent dental implant placements with straight parallel pins. **d** Coronal, (**e**) left lateral view and (**f**) postoperative CBCT scan images of the two adjacent dental implant placements without straight parallel pins. Blue and green lines represent the direction of each dental implant

**Fig. 2:**
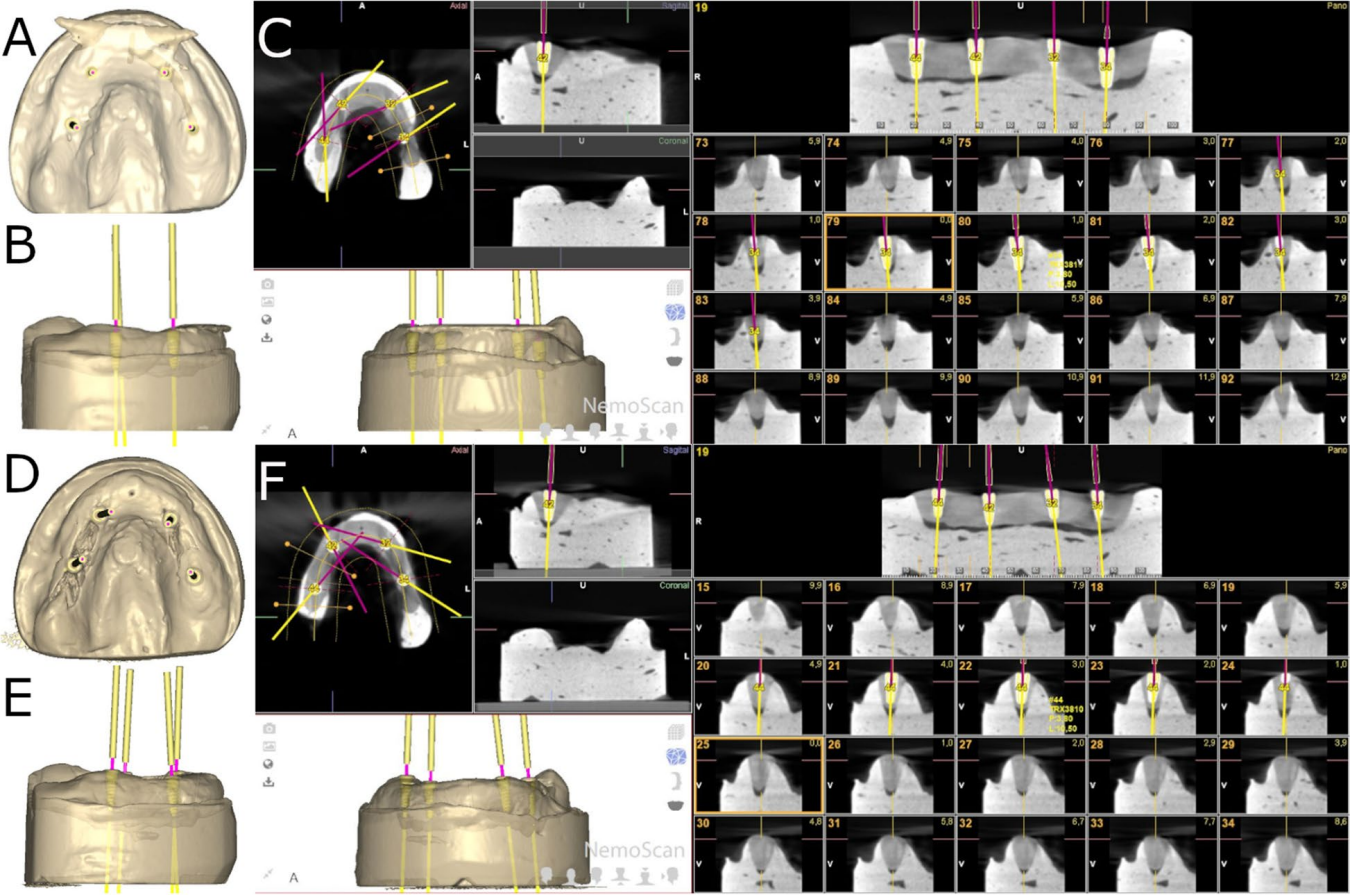
**a** Coronal, (**b**) left lateral view and (**c**) postoperative CBCT scan images of the four dental implant placements with straight parallel pins. **d** Coronal, (**e**) left lateral view and (**f**) postoperative CBCT scan images of the four dental implant placements without straight parallel pins. Yellow and pink lines represent the direction of each dental implant

**Fig. 3:**
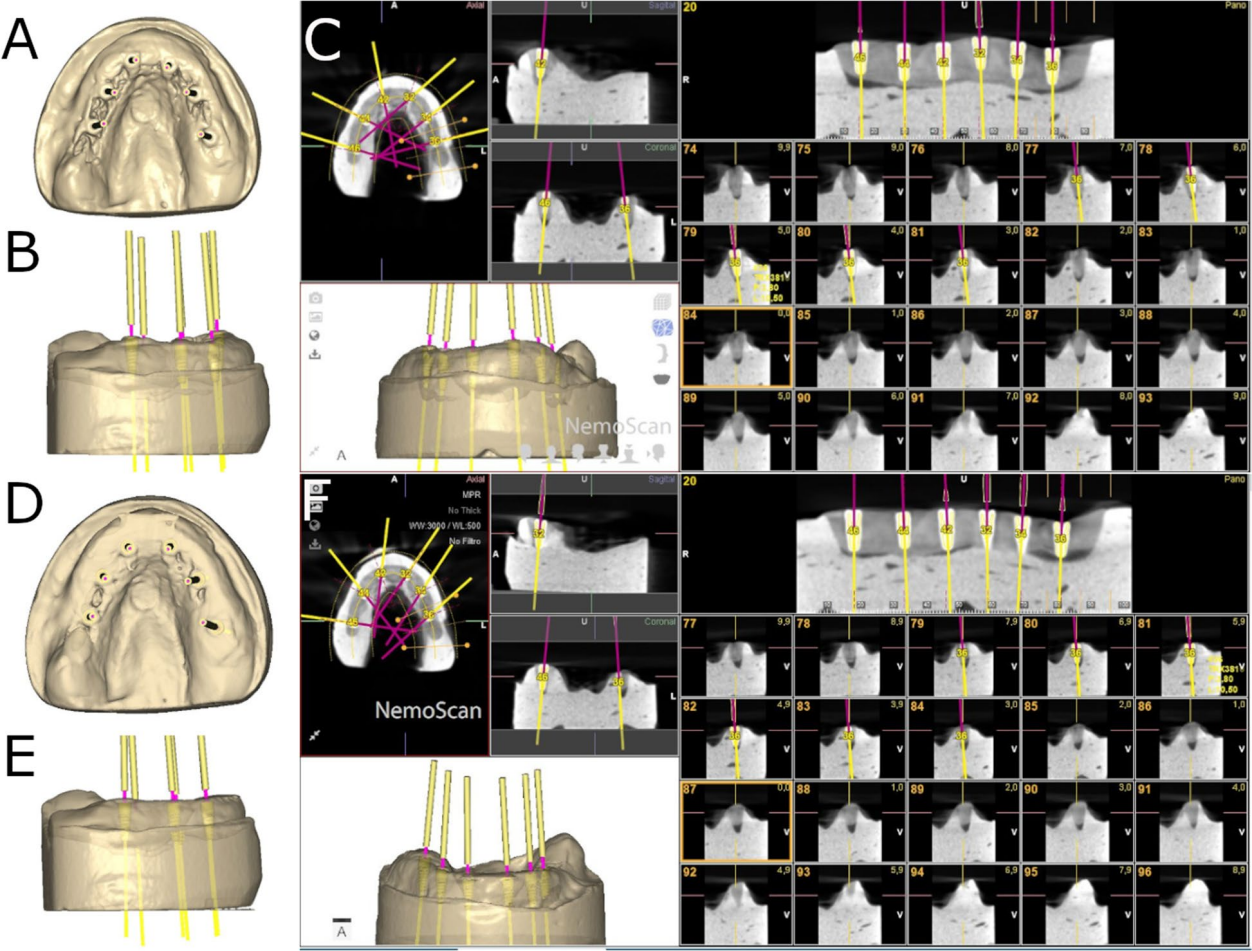
**a** Coronal, (**b**) left lateral view and (**c**) postoperative CBCT scan images of the six dental implant placements with straight parallel pins. **d** Coronal, (**e**) left lateral view and (**f**) postoperative CBCT scan images of the six dental implant placements without straight parallel pins. Yellow and pink lines represent the direction of each dental implant

The osteotomy site preparation of the dental implants randomly allocated to study groups 2PP (Fig. 1a-c), 4PP (Fig. 2a-c) and 6PP (Fig. 3a-c) were carried out freehand at 800 rpm with irrigation. The operator began by placing the posterior dental implant on the left side; Afterwards, a straight parallel pin was placed, and osteotomy site preparation was carried out in the anterior dental implant location and this procedure was continued sequentially until finishing with the placement of the posterior dental implant on the right side.

The osteotomy site preparation of the dental implants randomly allocated to study groups 2withoutPP (Fig. 1d-f), 4withoutPP (Fig. 2 d-f) and 6withoutPP (Fig. 3 d-f) were carried out freehand at 800 rpm with irrigation. The operator began by placing the posterior dental implant on the left side and continued to be placed sequentially until finishing with the placement of the posterior dental implant on the right side.

The following sequence of dental implant drills was used: 2.0–mm diameter dental implant drill (Ref.: TSD2020HD, BioHorizons, Birmingham, AL, USA), 2.5-mm diameter dental implant drill (Ref.: TSD2025HD, BioHorizons, Birmingham, AL, USA), 2.8-mm diameter dental implant drill (Ref.: TSD2028HD, BioHorizons, Birmingham, AL, USA), 3.2-mm diameter dental implant drill (Ref.: TSD2032HD, BioHorizons, Birmingham, AL, USA) and 3.7-mm diameter dental implant drill (Ref.: TSD2037HD, BioHorizons, Birmingham, AL, USA). The drills were inserted to a depth of 10 mm from the surface of the standardized polyurethane models of partially and fully edentulous upper jaws (Sawbones Europe AB, Malmo, Sweden).

The osteotomy site preparations and dental implant placement procedures for all study groups were manually performed in a phantom head by a unique operator with over 10 years’ experience in dental implant surgery, following the manufacturer’s recommendations. The operator was required to place the dental implants with 3 mm inter-implant distance [13], maintaining parallelism with the axial axis of adjacent teeth (groups A and B) and to a depth of the alveolar ridge to standardize the dental implant placement position.

### Measurement procedure

**Fig. 4:**
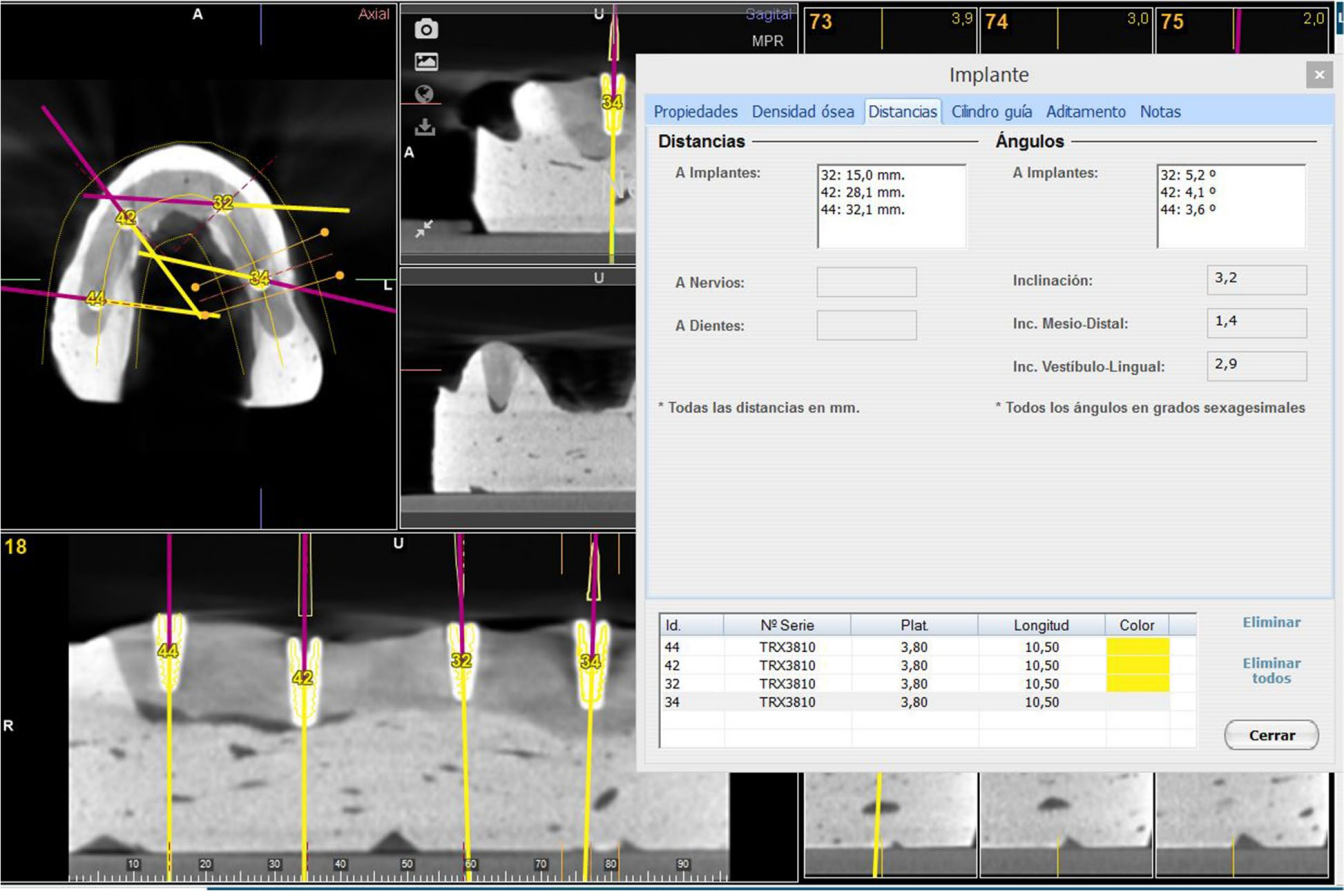
Illustration of the measurement procedure at the 3D implant-planning software

Postoperative CBCT scans were taken with the following exposure parameters: 105.0 kV peak, 8.0 mA, 7.20 s, and a field of view of 110 mm × 60 mm. Afterwards, the postoperative “Digital Imaging and Communication In Medicine” (DICOM) datasets from the CBCT scans were subsequently imported into 3D implant-planning software (NemoScan, Nemotec, Madrid, Spain). Then, the STL digital file from the preoperative planning and DICOM files were aligned to assess the angular deviation across the axial axis of all dental implants, measured in the center of the dental implants (Fig. 4). An independent operator evaluated all deviations across all implants and compared them in the axial, sagittal, and coronal views using the STL-data from the scan. The measurement procedure was performed by positioning an axis along the longitudinal axis of each dental implant that passed through the center of the platform and apex of the dental implant, and the angulation between the axes of each implant was measured in the 3D implant-planning software (NemoScan, Nemotec, Madrid, Spain).

### Statistical analysis

Statistical analysis of all variables was conducted in SAS 9.4 (SAS Institute Inc., Cary, NC, USA). Mean and standard deviation (SD) of the angulation between all dental implants of each study group were used to express descriptive statistics for quantitative variables and qualitative variables were expressed as absolute numbers and percentages. A General Linear Model statistical analysis was carried out to analyze the mean and deviation depending on the number of dental implants, the effect of the straight parallel pin and the interaction between both variables. In case of detecting statistically significant differences between times, 2 to 2 comparisons were made. The variables number of implants, straight parallel pins and the interaction number of implants*straight parallel pins were included as fixed factors in the model. The statistically significant result of the interaction indicates that the effect of the number of implants when pins were included is not the same as when the pins were not included. The *p*-values were adjusted with the Tukey method to correct the type I error. Statistical significance was set at *p* < 0.05.

## Results

**Fig. 5:**
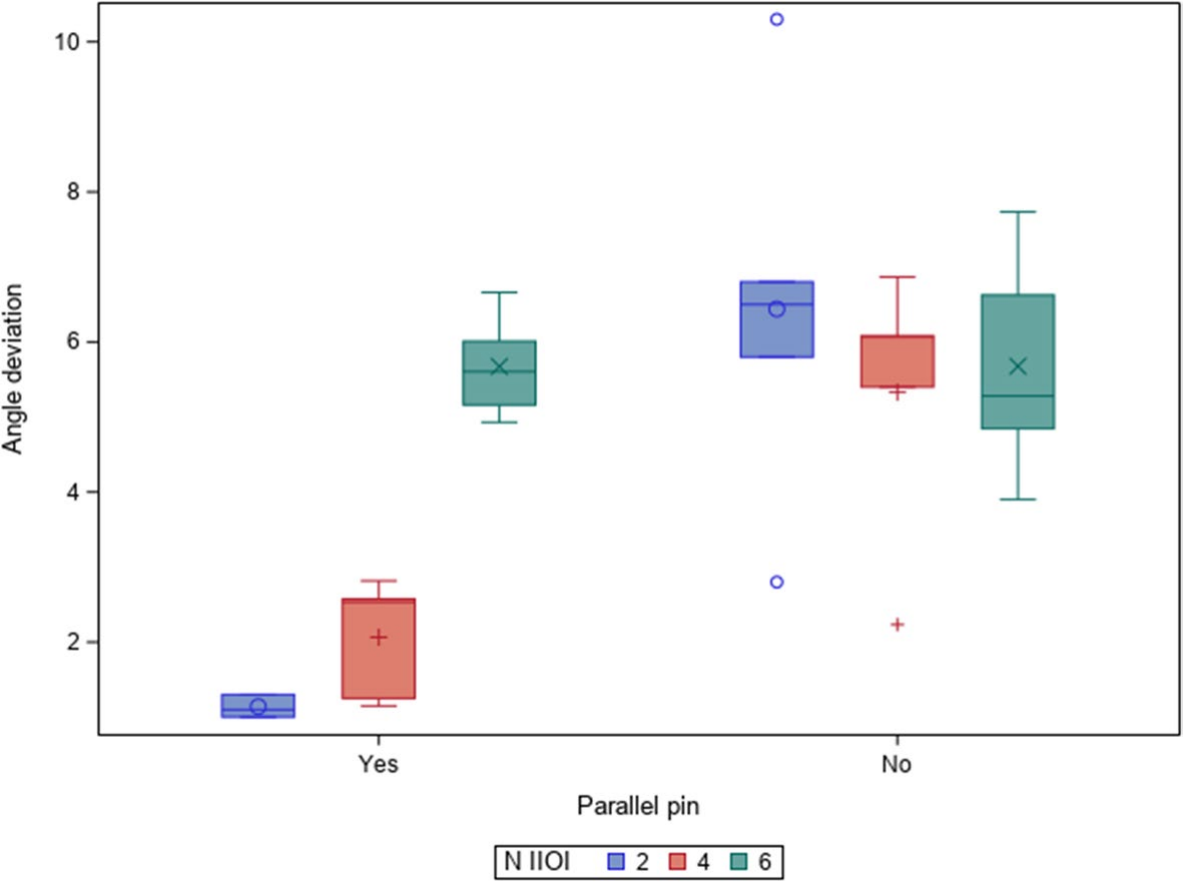
Box plots of the angle deviations observed in the experimental groups. The median values are represented by horizontal lines in each box. x, o and + expressed atypical values

Table 1 and Fig. 5 display the means and SD values of the angle deviation (°) between two, four and six adjacent dental implants placed with and without straight parallel pins.

**Table 1:** Descriptive deviation values angulation (°) between all dental implants of each study group

Study Group	***n***	Mean	SD	Minimum	Maximum
2PP	10	1.14	0.15	1.00	1.30
2withoutPP	10	6.44	2.68	2.80	10.30
4PP	10	2.07	0.80	0.15	2.82
4withoutPP	10	5.33	1.81	2.23	6.87
6PP	10	5.67	0.69	4.93	6.66
6withoutPP	10	5.68	1.51	3.90	7.73

Statistically significant differences were shown at the angle deviation (°) between the number of dental implants (*p =* 0.0117), the use of straight parallel pins (*p <* 0.0001) and between the interaction of the number of dental implants and the use of straight parallel pins *(p =* 0.0026) (Fig. 5).

In addition, Student’s *t*-test showed statistically significant differences between the mean angle deviation (°) of 2 and 6 dental implants *(p =* 0.0275) and between the mean angle deviation (°) of 4 and 6 dental implants *(p =* 0.0203); however, no statistically significant differences were shown between the mean angle deviation (°) of 2 and 4 dental implants *(p =* 0.9898) (Fig. 5).

Furthermore, the mean angle deviation was 2.8° higher between the dental implants placed without a straight parallel pin.

In summary, the use of a straight parallel pin resulted in statistically significant differences between the angle deviation (°) of dental implants (*p =* 0.0002); however, the dental implants placed without a straight parallel pin did not show statistically significant differences in angle deviation (°) (*p =* 0.5075). Specifically, statistically significant differences were shown between the angle deviation (°) of 2 dental implants placed with a straight parallel pin (*p <* 0.0001) and between the angle deviation (°) of 4 dental implants placed with a straight parallel pin (*p =* 0.0024); however, no statistically significant differences were shown between the angle deviation (°) of 6 dental implants placed with a straight parallel pin (*p =* 0.9967) (Fig. 5).

## Discussion

The results of the present study rejected the null hypothesis (H_0_) that there is no difference between the angle deviation of adjacent dental implants placed with a straight parallel pin and the angle deviation of the adjacent dental implants placed without a straight parallel pin.

The results of this study recommend the use of the parallelization pin to avoid angular deviations during dental implant placement, especially for the placement of a reduced number of dental implants (between 2 and 4), as it does not appear to be useful after 6 dental implants. It was observed that the angulation between two dental implants placed with a straight parallel pin statistically improved the parallelism between them, comparing with the two dental implants placed without a straight parallel pin. These results were also shown among the 4 implants placed with and without the aid of the paralleling pin. However, the use of the parallelization pin was not useful in the degree of parallelism of 6 implants, probably because it is it is difficult to analyze the parallelism between the implants that are further away despite having the help of the parallelization pin.. Additionally, Ramaray et al. reported a case report where a straight parallel pin was accidentally ingested; therefore, recommendations to avoid instrument ingestion should must be taken into account [14], such as placing a gauze pad at the posterior aspect of the mouth or using a dental floss [15] Flanagan D (2018) reported that parallelism between dental implants in curved edentulous dental arch may be impossible [16]; however, Renouard et Rangert highlighted that non-parallel placement may better resist occlusal loads [17] and there are prosthetic fabrication techniques to correct dispallalelism between dental implants [1].

The present study showed less influence of the parallel pin on fully edentulous upper jaws; however, Gilizio et al. (2005) highlighted the parallelism in removable overdentures for the fabrication accuracy and to prevent wear issues of the retainers [18] and Al-Ghafli et al. reported that mplant angulations negatively affect attachment retention longevity [19]. Therefore, computer-aided implant surgery through static and dynamic navigation systems will be recommended for the dental implant placement in fully edentulous upper jaws.

Conventional implant impression procedures are conducted by recording implant position using impression coping, elastomeric material, and a rigid tray. Several factors that influence the accuracy of conventional impression have been identified in the literature, including number of implants, angulation, depth, impression technique, and impression material [20–22]. Barjani et al. emphasized the significant impact of implant angulation on impression taking errors (*p* = 0.0001) [23]. Carr [24] and Assuncao et al. [25] found that angulated implants had lower dimensional accuracy than straight implants and Conrad et al. concluded that when a greater number of implants with different angulation is used, the impression material undergoes more dimensional changes [26]. However, Choi et al. evaluated the accuracy of two impression techniques for parallel or 8° divergent internal hexagonal dental implants, finding that both impression techniques had the same level of accuracy, and a divergence of up to 8° did not significantly affect the accuracy of the impression techniques [27]. In addition, Ribeiro et al. compared the accuracy of dental impressions taken digitally with those made using conventional techniques, analyzing both parallel dental implants and non-parallel dental implants; they found that the open tray technique used for non-parallel dental implants impression provided a median of the square (1,257,835) significantly lower (*p* < 0. 001) than the impression taken with a closed tray (1,660,975) and the digital impression technique (1,489,328) at the X-deviation, Y-deviation, and Z-deviation. However, the digital impression technique used for parallel dental implants showed a lower median of the square (1,068,292) than the impression taken with a closed tray (2,114,342) and the impression taken with an open tray technique (2,165,491) and the at the X-deviation, Y-deviation, and Z-deviation. This study was also performed in fully edentulous upper jaws by placing four dental implants in each [28]. These results were corroborated by Abduo et al., who reported that the digital impression techniques provided greater accuracy in terms of precision, trueness, and angle deviation; however, this study was performed in a partial master model with two dental implants [29].

Biomechanically, implant-supported restorations are manufactured with the goal of ensuring a passive fit of abutments and fixtures in the attachment; this is imperative to ensure equal distribution of stress at the bone–implant interface. A misfit leads to internal stresses, which subsequently transfer to implants and the bone matrix [30]. Evidence also indicates that a lack of passive fit in implant-supported restorations can lead to tiny bone fractures or ischemic marginal zones. When these lesions heal, they form fibrous connective tissue at the bone–implant interface that can prevents osseointegration or lead to peri-implant bone loss [31]. An ill-fitting framework can also trigger biological and biomechanical problems. Applying excessive load that exceeds the natural threshold of the implant-supporting bone can lead to pain, marginal bone loss, tissue irritation, and impaired osseointegration [26, 32]. In addition, implant components may have issues with biomechanical problems such as mobility and fractures [33, 34]. An active fit is the primary cause of loosening of abutment screws, mobility of restorations, bone loss, and fractured implant components [35]. The angulation of dental implants significantly affects (*p* = 0.0001) the dimensional accuracy of the master cast and therefore the passive fit of prosthetic frameworks, with an angle deviation of 25° between two adjacent dental implants showing the lowest accuracy (R = 1.1336) [23].

Due to its experimental nature, this in vitro study is somewhat limited in scope. However, the study methodology can easily be applied to clinical studies in order to provide evidence of the effect of parallel pins on the angle deviation of dental implants, particularly in a reduced number of dental implants.

## Conclusions

In conclusion, bearing in mind the limitations of this study, the results indicate that straight parallelization pins result in less angle deviation between two and four adjacent dental implants; however, they are not effective for a larger number of dental implants.

## Data Availability

The datasets used and/or analyzed in the present study are available from the corresponding author upon reasonable request.
